# *Lactobacillus acidophilus* attenuates *Salmonella*-induced intestinal inflammation via TGF-β signaling

**DOI:** 10.1186/s12866-015-0546-x

**Published:** 2015-10-07

**Authors:** I-Fei Huang, I-Chun Lin, Pei-Feng Liu, Ming-Fang Cheng, Yen-Chen Liu, Yao-Dung Hsieh, Jih-Jung Chen, Chun-Lin Chen, Hsueh-Wei Chang, Chih-Wen Shu

**Affiliations:** Department of Pediatrics, Kaohsiung Veterans General Hospital, Kaohsiung, Taiwan; Faculty of Medicine, National Yang-Ming University, Taipei, Taiwan; Chung Hwa University of Medical Technology, Tainan, Taiwan; Diet and Nutrition Section, Zuoying Branch of Kaohsiung Armed Forces General Hospital, Kaohsiung, Taiwan; Department of Medical Education and Research, Kaohsiung Veterans General Hospital, Kaohsiung, Taiwan; Department of Dentistry, Kaohsiung Veterans General Hospital, Pingtung, Taiwan; Department of Pharmacy, Tajen University, Pingtung, Taiwan; Department of Biological Science, National Sun Yat-sen University, Kaohsiung, Taiwan; Doctoral Degree Program in Marine Biotechnology, National Sun Yat-sen University and Academia Sinica, Kaohsiung, Taiwan; Department of Biomedical Science and Environmental Biology, Kaohsiung Medical University, Kaohsiung, Taiwan; Research Center of Environmental Medicine, Kaohsiung Medical University, Kaohsiung, Taiwan

**Keywords:** *L. acidophilus*, Synbiotics, *Salmonella*, TGF-b, NF-κB, MIR21, SMAD

## Abstract

**Background:**

*Salmonella* is a common intestinal pathogen that causes acute and chronic inflammatory response. Probiotics reduce inflammatory cytokine production and serve as beneficial commensal microorganisms in the human gastrointestinal tract. TGF-β (transforming growth factor β)/SMAD and NF-κB signaling play important roles in inflammation in intestinal cells. However, the involvement of the signaling in regulating inflammation between *Salmonella* and probiotics is not fully understood.

**Methods:**

L. *acidophilus* and prebiotic inulin were used to treat human intestinal Caco-2 cells prior to infection with *Salmonella*. The cells were harvested to examine the cytokines and MIR21 expression with immunoblotting and real-time PCR. NF-κB and SMAD3/4 reporter vectors were transfected into cells to monitor inflammation and TGF-β1 signaling, respectively.

**Results:**

In this study, we showed that the probiotic *L. acidophilus* decreased *Salmonella-*induced NF-κB activation in human intestinal Caco-2 cells. Expression of the inflammatory cytokines, TNF-α and IL-8, in *L. acidophilus*-pretreated cells was also significantly lower than that in cells infected with *Salmonella* alone. Moreover, TGF-β1 and MIR21 expression was elevated in cells pretreated with *L. acidophilus* or synbiotic, a combination of inulin and *L. acidophilus*, compared to that in untreated cells or cells infected with *S. typhimurium* alone. By contrast, expression of SMAD7, a target of MIR21, was accordingly reduced in cells treated with *L. acidophilus* or synbiotics. Consistent with TGF-β1/MIR21 and SMAD7 expression, SMAD3/4 transcriptional activity was significantly higher in the cells treated with *L. acidophilus* or synbiotics. Furthermore, TGF-β1 antibody antagonized the SMAD3/4 and NF-κB transcriptional activity modulated by *L. acidophilus* in intestinal cells.

**Conclusion:**

Our results suggest that the TGF-β1/MIR21 signaling pathway may be involved in the suppressive effects of *L. acidophilus* on inflammation caused by *S. typhimurium* in intestinal Caco-2 cells.

**Electronic supplementary material:**

The online version of this article (doi:10.1186/s12866-015-0546-x) contains supplementary material, which is available to authorized users.

## Background

The human intestine houses a dense and diverse microbial community, which is estimated to contain at least 500 different species of bacteria. These microbes, collectively referred to as the commensal microbiota, play important roles in human health, such as processing of nutrients, regulation of fat storage [1, 2], and protection against pathogens [3]. *Salmonella typhimurium* (*S. typhimurium*), an intestinal pathogenic bacterium, is one of the most common non-typhoidal *Salmonella* (NTS), which is the leading cause of acute food-borne disease [4]. Salmonellosis results in diarrhea, vomiting, and fever in the majority of people after infection. Approximately 400 persons die each year because of severe Salmonellosis, particular young children, the elderly, and the immunocompromised persons. *S. typhimurium* penetrates intestinal cells and macrophages to induce intestinal inflammation using two type III secretion systems (T3SSs) [5]. *S. typhimurium* induces NF-kB activation and the secretion of pro-inflammatory cytokines, such as interleuckin-8 (IL-8) [6] and tumor necrosis factor alpha (TNF-α) [7]. The excessive inflammatory response may help *S. typhimurium* eliminate other competitive microbiota in the host [8].

Probiotics attenuate NF-κB activation and inflammatory cytokine production in intestinal epithelia cells [9, 10] and *in vivo* [11–13]. These probiotics are considered beneficial commensal microorganisms in human gastrointestinal tract [14, 15]. TGF-β was identified as a negative regulator of NF-κB activation in gut inflammation [16]. TGF-β activates SMAD2/3 and initiates SMAD4 dependent transcription to induce IκBα, inhibitor of NF-κB, and attenuate inflammatory responses [16], whereas SMAD7 physically inhibits the TGF-β receptor and induces the pro-inflammatory pathways mediated by NF-κB [17]. Our previous study showed that probiotics enhance enteric protection against pathogens and reduce mucosal inflammation by enhancing tumor growth factor beta (TGF-β) signaling *in vivo* [18]. Recent reports also demonstrate that TGF-β induces microRNA-21 (MIR21) expression, which targets 3'-UTR of SMAD7 to attenuate its expression and enhances TGF-β signaling [19–21]. Furthermore, probiotics decrease SMAD7 expression and elevate IκBα expression to suppress pathogen-induced inflammation in human gastric epithelia cells [9], suggesting that feed-back control between TGF-β and SMAD7 may be crucial for inflammation.

Although several studies demonstrated the suppression of inflammation using probiotics, the TGF-β-mediated signaling caused by probiotics administration on *S. typhimurium*-induced inflammation in human intestinal cells remains unclear. In this study, we examined the role of TGF-β signaling molecules in human intestinal Caco-2 cells infected with *S. typhimurium* in the absence or presence of the probiotic *L. acidophilus* alone or combination with the prebiotic inulin, a non-digestible oligosaccharide that enhances the growth of probiotics. We found that TGF-β and MIR21 expression is induced, whereas SMAD7 expression was decreased in human colorectal Caco-2 cells pretreated with *L. acidophilus* compared to cells infected with *S. typhimurium* alone*.* SMAD3/4 transcriptional activity was further elevated in *L. acidophilus*-treated cells, consistent with reduced NF-kB transcriptional activity and inflammatory cytokines induction, including IL-8 and TNF-α. Therefore, our findings elucidate the effect of TGF-β signaling on probiotics or synbiotics, which may identify a new treatment strategy for inflammation caused by *Salmonella* infection using probiotics.

## Methods

### Cell culture

The human colonic adenocarcinoma cell line (Caco-2, BCRC 60182) was used in this study and cultured in Dulbecco’s modified Eagle’s medium (DMEM) (Invitrogen, 12100–046) supplemented with 3.7 g/L NaHCO3 (Merck), 1.7 mM glutamine (Brunschwig Chemie BV), 100 U/ml penicillin, 100 μg/ml streptomycin, 0.25 μg/ml amphotericin (Biological Industries), 0.01 mg/ml human transferrin (T-0665, Sigma-Aldrich), 1.0 mM sodium pyruvate, and 10 % (v/v) fetal bovine serum (Biological Industries) at 37 °C in a humidified atmosphere of 5 % (v/v) CO_2_ in air. Cells were seeded in 75 cm^2^ tissue culture flasks (Corning Incorporated) containing 8 ml DMEM medium. This study encompassed 10 cell passages of the cell line ranging from the 20th to the 29^th^ passage.

### *S. typhimurium* culture and infection

The enteric pathogen *S. typhimurium* (BCRC 10747) was grown on a blood agar plate (BAP, trypticase soy agar with 5 % sheep blood) (CMP Ltd.) at 37 °C overnight. The bacterial colonies were collected with disposable L-shaped cell spreaders in PBS (Biological Industries). The collected bacteria were washed, centrifuged at 1,5000 × g for 15 min at 4 °C and resuspended in PBS. The concentration of *S. typhimurium* was measured using a spectrophotometer as the optical density (OD) at 600 nm. For bacterial infection, Caco-2 cells (1×10^6^ cells/well) were incubated with various concentrations *S. typhimurium*, ranging from 1 × 10^4^ CFU/ml to 1 × 10^10^ CFU/ml in antibiotic-free DMEM for 1 h at 37 °C. The cells were washed with PBS and recovered with DMEM media containing gentamicin (50 μg/ml, Sigma-Aldrich) for the following experiments.

### Treatment of probiotic *L. acidophilus*

The probiotic *L. acidophilus* (BCRC 10695) was cultured in DeMan, Rogosa, Sharpe (MRS) broth (CMP Ltd.) at 37 °C overnight. After centrifugation at 15000 × g for 15 min at 4 °C, the collected bacteria were washed once with PBS (Biological Industries, 02-023-1) and subsequently resuspended with PBS for the treatment of Caco-2 cells. Caco-2 cells (1×10^6^ cells/well) were seeded in a 6-well plate and treated with *L. acidophilus* (2 × 10^7^ CFU/ml/well, MOI = 20) in the presence or absence of prebiotic (1 % inulin, Orafti ®GR) or probiotic in antibiotic-free DMEM for 1 h. Subsequently, the Caco-2 cells were infected with *S. typhimurium* (1 × 10^7^ CFU) for 1 h and recovered with DMEM media containing gentamicin (50 μg/ml, Sigma-Aldrich) for the following experiments.

### Transfection and luciferase reporter assay

To test the SMAD and NF-κB transcriptional activity, the Caco-2 cells in a 384-well plate were transfected with 50 ng of plasmid containing NF-κB-responsive reporter vector pGL4.32 (Promega) or SMAD responsive reporter vector pGL4.48 (Promega), which contains the NF-κB and SMAD-binding sites, respectively. The pGL4.50 (Promega) constitutively expressing luciferase was used as a normalization control. At 20 h post transfection, the cells were infected with *S. typhimurium* in the absence of presence of probiotic or synbiotic for 1 h. The cells were washed once with PBS and recovered with DMEM media containing gentamicin (50 μg/ml, Sigma-Aldrich). The luminescence of the luciferase-based reporter was measured with DMEM medium containing 200 μM D-luciferin (Promega). The results were read using a Fluoroskan Ascent FL reader (Thermo Fisher Scientific).

### Protein extraction and western blot

Human colorectal epithelia Caco-2 cells were briefly rinsed in PBS (Biological Industries) and lysed with RIPA buffer (1 % NP40 [MDBio, 101-9016-45-9], 50 mM Tris HCl, pH 7.5, 150 mM NaCl, 0.25 % sodium deoxycholate [Sigma-Aldrich, D6750], 0.1 % sodium dodecyl sulfate [SDS; Calbiochem, 428015], and protease inhibitor cocktail [Roche, 11873580001]). The cell lysates were centrifuged at 13200 × g for 30 min and the supernatant was collected to determine the protein concentration using Bradford Reagent (Sigma-Aldrich). The cell lysates were resolved by SDS-PAGE and transferred onto nitrocellulose membranes using a wet electrophoretic transfer system (Bio-Rad). The membrane was blocked with 5 % skim milk and then incubated with primary antibodies against SMAD7 (sc-11392) (Santa Cruz Biotech), TGF-β (abcam) and actin (Sigma-Aldrich) to determine the protein expression using the ChemiDoc XRS Imaging System (Bio-Rad) and analysis with Image Lab4.1 (Bio-Rad).

### Real-Time PCR

The cells transfected with siRNA were used to extract the total RNA with TRIzol Reagent (Invitrogen, 15596–018). A total of 1 μg RNA was reverse-transcribed with SuperScript II RNase H-Reverse Transcriptase (Invitrogen, 18064–014) for cDNA synthesis. The amounts of IL-8, TNF-α and MIR21 mRNA relative to S-26 were analyzed by real-time PCR performed in a StepOnePlus^™^ system (Applied Biosystems) with the SYBR Green Master Mix (Applied Biosystems, 4385612). The primers for the genes are as follows: IL-8 forward 5′- ACTGAGAGTGATTGAGAGTGGAC-3′ and reverse 5′- AACCCTCTGCACCCAGTTTTC −3′, TNF-α forward 5′- GAGGCCAAGCCCTGGGATG −3′ and reverse 5′- CGGGCCGATTGATCTCAGC −3′, S-26 forward 5′- CCGTGCCTCCAAGATGACAAAG −3′ and reverse 5′- GTTCGGTCCTTGCGGGCTTCAC −3′, and MIR21 reverse transcription 5′- CTCAACTGGTGTCGTGGAGTCGGCAATTCAGTTGAGTCAACATC −3′ and gene specific forward 5′- CGGCGGTAGCTTATCAGACTGA −3′.

### Data analysis

Data were statistically analyzed by Prism 5.0 (Graph-Pad) using one-way ANOVA followed by Tukey’s multiple comparison test to compare the effects between each group. All results are expressed as the mean ± SEM from at least 3 individual experiments. The lev el of significance was set at *P* < 0.05 (*P*-value ≦ 0.05 considered significant (*), *P*-value ≦ 0.01 considered highly significant (**), and *P*-value ≦ 0.001 considered extremely significant (***)).

## Results

### *L. acidophilus* attenuates *S. typhimurium*-induced inflammatory response in Caco-2 cells

**Fig. 1:**
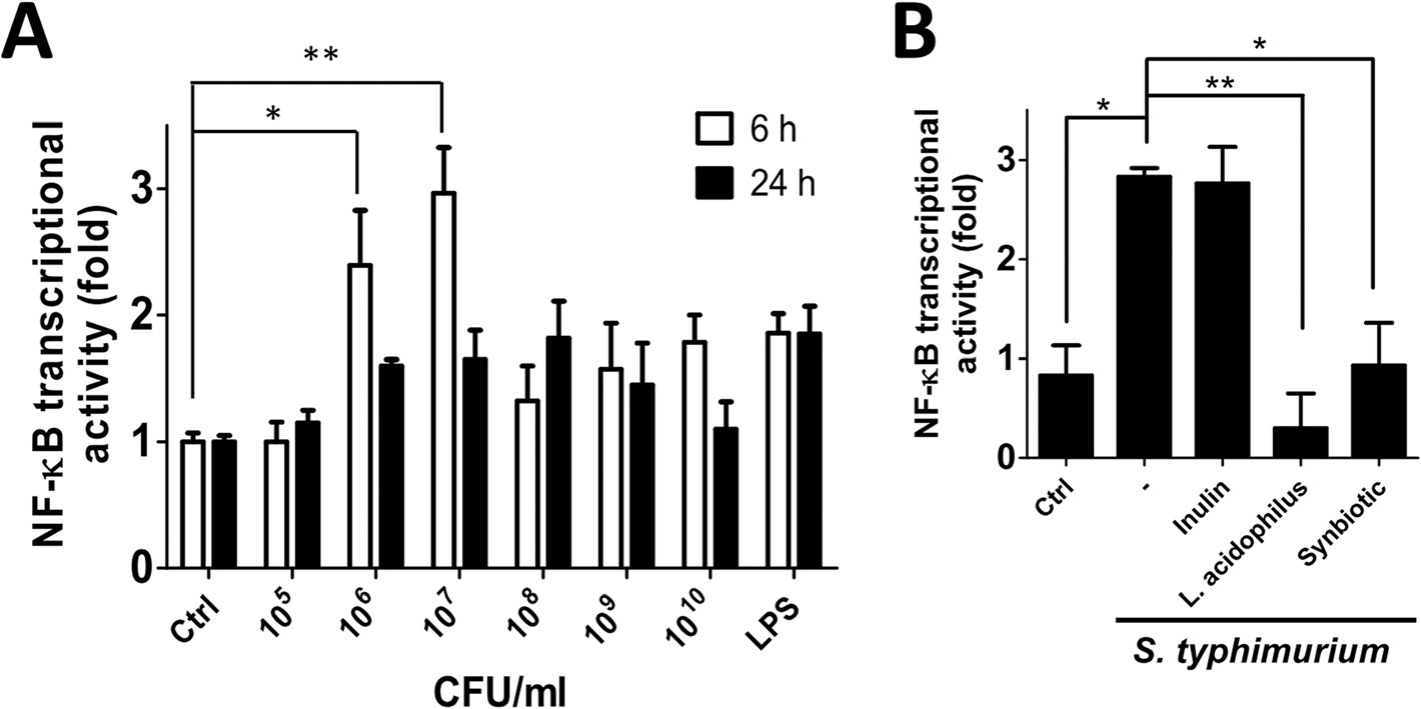
Effects of *L. acidophilus* on *S. typhimurium*-induced NF-κB activation. **a** Human intestinal Caco-2 cells were transfected with a luciferase reporter vector for NF-κB or CMV (normalization control) overnight and then infected with various concentrations of *S. typhimurium* as indicated, ranging from 10^4^ to 10^10^ CFU/ml in antibiotic-free DMEM for 1 h at 37 °C. The cells were recovered with DMEM media containing gentamicin (50 μg/ml) and NF-κB transcriptional activity was measured at 6 or 24 h post-infection. **b** For treatment with *L. acidophilus*, transfected cells were treated with 1 % inulin or *L. acidophilus* (*L. acidophilus*:*S. typhimurium* = 2:1) or synbiotics (*L. acidophilus* and 1 % inulin) 1 h prior to infection of *S. typhimurium* as mentioned above. The cells were added with DMEM medium containing D-luciferin and antibiotic at 6 h post-infection to measure NF-κB activity. The data were analyzed with Prism 5, and the results are shown as the means ± SEM from three independent experiments

**Fig. 2:**
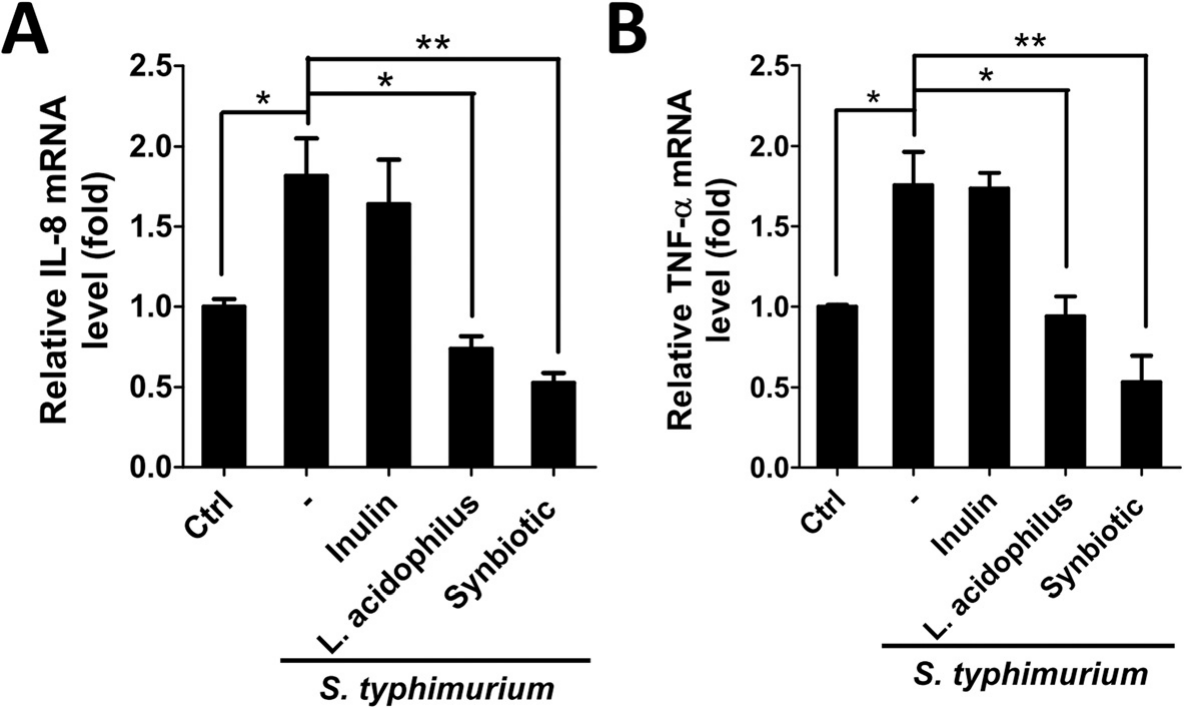
Effects of *L. acidophilus and S. typhimurium* on IL-8 and TNF-α mRNA expression. Human intestinal Caco-2 cells were treated with 1 % inulin or *L. acidophilus* (*L. acidophilus*:*S. typhimurium* = 2:1) or synbiotics (*L. acidophilus* and 1 % inulin) 1 h prior to infection with *S. typhimurium* in DMEM without antibiotic for 1 h. The cells were harvested at 6 h post-infection for mRNA isolation. The isolated mRNA was further used to determine the expression of (**a**) IL-8 and (**b**) TNF-α by quantitative PCR (qPCR). The data are shown as the means ± SEM from three independent experiments

NF-κB transcribes global genes involved in inflammation, such as TNF-α [22] and IL-8 [23]. To explore the effect of probiotic on *S. typhimurium*-induced inflammation, an NF-κB responsive reporter assay was used to optimize the infected concentration of *S. typhimurium* for human intestinal cells. Human intestinal Caco-2 cells were transfected with NF-κB responsive reporter vector for 16 h and then incubated with *S. typhimurium*, ranging from 1 × 10^6^ ~ 1 × 10^10^ CFU/ml (MOI 1 to 10000), in antibiotic-free media for 1 h (Fig. 1). The infected Caco-2 cells were recovered with media containing gentamicin to monitor NF-κB transcriptional activity at 6 h or 24 h post-infection. NF-κB transcriptional activity was significantly increased 2.5- and 3-fold in Caco-2 cells infected with *S. typhimurium* at 6 h post-infection in a concentration of 1 × 10^6^ CFU/ml (*p*≦ 0.05) and 1 × 10^7^ CFU/ml (*p*≦ 0.01), respectively (Fig. 1a). Therefore, the optimal ratio for *S. typhimurium* to human colorectal epithelia Caco-2 cells is an MOI 10 for this study. Moreover, *S. typhimurium* entered host cells at 3 h after infection (Additional file 1: Figure S1), suggesting that *S. typhimurium* triggered inflammatory response may be mainly resulted from extracellular stimuli, most likely are the lipopolysaccharides of *S. typhimurium*. Because *L. acidophilus* is one of the most commonly used probiotics in the food supply, we used *L. acidophilus* as a probiotic for the following experiments. Further, inulin is a well-known prebiotic that is used to improve the growth and viability of probiotics in fermented milk [24]. Therefore, we employed human intestinal Caco-2 cells harboring NF-κB responsive reporter vector to infect with *S. typhimurium* (MOI 10) without or with inulin or *L. acidophilus* (MOI 20) or combination (synbiotic). Both *L. acidophilus* (MOI 20) and synbiotic significantly suppressed NF-κB transcriptional activity in cells infected with *S. typhimurium* (*p*≦ 0.05) (Fig. 1b), whereas inulin alone did not alter *S. typhimurium*-induced NF-κB transcriptional activity. Furthermore, the mRNA level of IL-8 and TNF-α, inflammatory cytokines and transcripts of NF-κB, were significantly reduced in *L. acidophilus* and synbiotic pretreated Caco-2 cells compared to the cells infected with *S. typhimurium* alone (Fig. 2) (*p*≦0.05), suggesting that the *L. acidophilus* and synbiotic decrease NF-kB activation, which in turn inhibits inflammatory cytokine induction and reduces the inflammation in human intestinal cells during *S. typhimurium* infection.

### *L. acidophilus* induces TGF-β and MIR21 expression

**Fig. 3:**
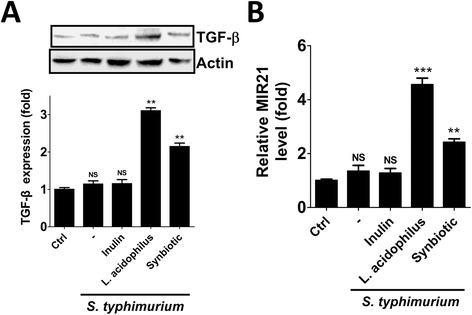
Effects of *L. acidophilus* and *S. typhimurium* on TGF-β and MIR21 expression. Human intestinal Caco-2 cells were treated with 1 % inulin or *L. acidophilus* (*L. acidophilus* : *S. typhimurium* = 2:1) or synbiotics 1 h prior to infection with *S. typhimurium* in DMEM without antibiotic for 1 h. The cells were harvested at 6 h post-infection for protein extraction. **a** The cells were lysed to extract the proteins and assess TGF-β expression by immunoblotting. **b** The treated cells were harvested at 6 h post-infection for total RNA isolation. The isolated RNA was further used to determine the expression of MIR21 by quantitative PCR (qPCR). The results are from three independent experiments, and the data are shown as the means ± SEM. NS: not significant

TGF-β, an important suppressor of inflammation, is induced by *L. acidophilus* treatment in a mouse model [18], whereas TGF-β expression did not change in human gastric epithelial cells [9]. The role of TGF-β in *L. acidophilus*-treated human colorectal epithelia Caco-2 cells is not fully understood. To determine whether TGF-β is involved in the anti-inflammation of *L. acidophilus* in Caco-2 cells during *S. typhimurium* infection, TGF-β expression in treated cells was assessed by immunoblotting (Fig. 3a). Both *L. acidophilus* and synbiotic increased TGF-β expression in human intestinal Caco-2 cells during *S. typhimurium* infection. Moreover, TGF-β enhances MIR21 maturation in primary cultured fibroblasts [19]; therefore, we further evaluated MIR21 expression in the treated Caco-2 cells (Fig. 3b). Consistently, MIR21 expression was increased in cells with *L. acidophilus* or synbiotic, but not in the other treated cells, suggesting that the TGF-β/MIR21 axis may be involved in the anti-inflammatory effects of *L. acidophilus* in Caco-2 cells post-infected with *S. typhimurium*.

### *L. acidophilus* increases TGF-β/SMAD signaling in intestinal cells

**Fig. 4:**
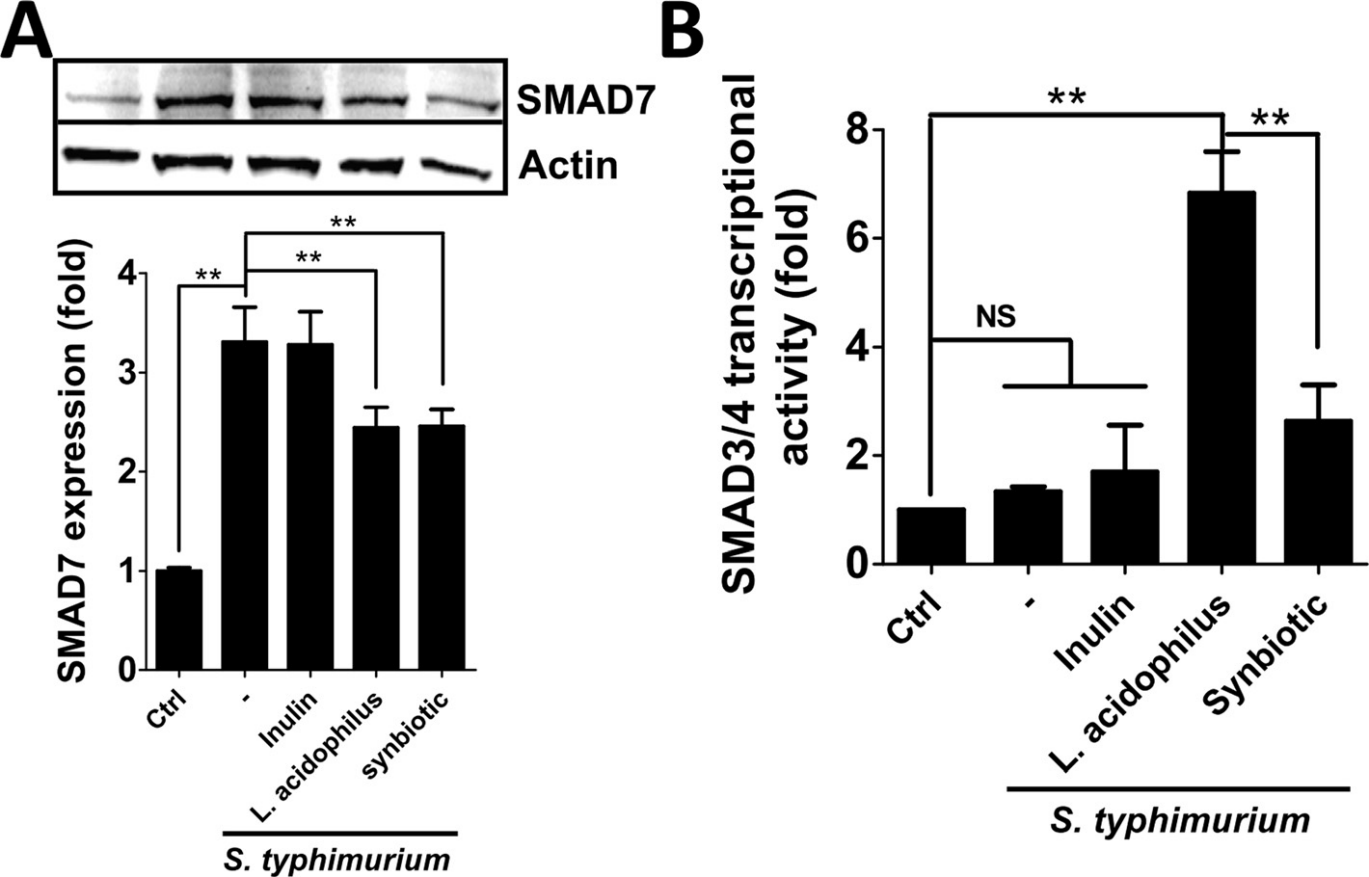
Effects of *L. acidophilus* and *S. typhimurium* on SMAD7 expression and SMAD3/4 transcriptional activity. **a** Human intestinal Caco-2 cells were treated with 1 % inulin or *L. acidophilus* (*L. acidophilus*:*S. typhimurium* = 2:1) or synbiotics (*L. acidophilus* and 1 % inulin) 1 h prior to treatment with *S. typhimurium* in DMEM without antibiotic for 1 h. The cells were harvested at 6 h post-infection for protein extraction. SMAD7 protein expression was determined by immunoblotting. **b** Human intestinal Caco-2 cells were transfected with luciferase reporter plasmid for SMAD3/4 or CMV (normalization control) overnight and then treated or infected as above. The cells were added DMEM medium containing D-luciferin at 6 h post-infection to read the signal with a Luminometer. The quantitative results are shown as the means ± SEM from three independent experiments. NS: not significant

**Fig. 5:**
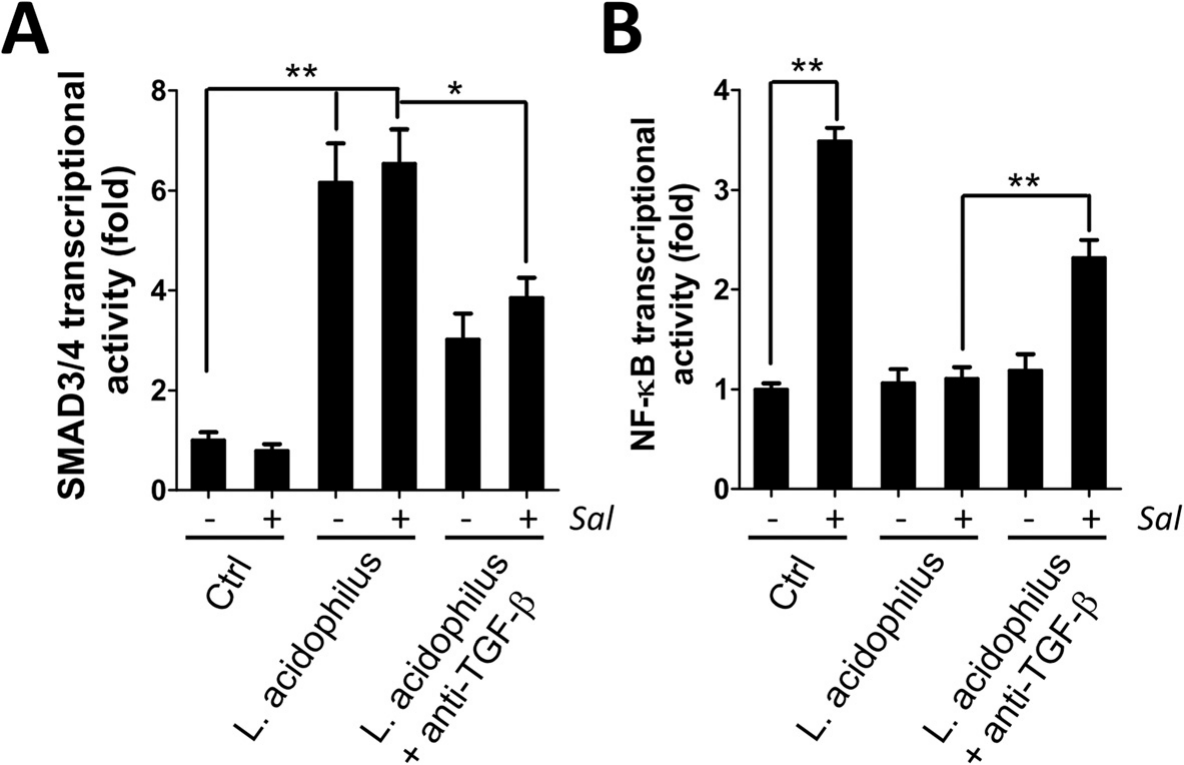
Effects of TGF-β on *L. acidophilus* regulated SMAD3/4 and NF-κB transcriptional activity. Human intestinal Caco-2 cells were transfected with luciferase reporter plasmid for **a** SMAD3/4 or **b** NF-κB overnight and then treated with *L. acidophilus* (*L. acidophilus*:*S. typhimurium* = 2:1) or *L. acidophilus* mixed with anti-TGF-β antibody (1 μg/ml) 1 h prior to infection with *S. typhimurium*. The cells were recovered and added DMEM medium containing D-luciferin at 6 h post-infection to read the signal with a Luminometer. The results are normalized with the cells harboring luciferase constitutively expressed vector (CMV) and are shown as the means ± SEM from three independent experiments

MIR21 targets 3'-UTR of SMAD7 and attenuates gene expression [19], which may augment TGF-β signaling for SMAD3/4 transcriptional activity, thereby playing an important role in this positive feedback loop to suppress inflammation. The protein expression of SMAD7 in *S. typhimurium* infected cells with or without *L. acidophilus* was determined by immunoblotting (Fig. 4a). The expression of SMAD7 was significantly increased in cells infected with *S. typhimurium* compared to the uninfected cells*,* whereas the cells pretreated with *L. acidophilus* or synbiotic had a reduction in the increased expression of SMAD7 (Fig. 4a). We further evaluated the SMAD3/4 transcriptional activity with the cells harboring SMAD3/4 responsive reporter vector. *L. acidophilus* induced SMAD3/4 activity in intestinal cells with either live or UV-inactivated *S. typhimurium* or LPS (Additional file 2: Figure S2). Moreover, SMAD3/4 transcriptional activity was increased 7-fold and 2.5-fold in cells administered with *L. acidophilus* and synbiotics, respectively (Fig. 4b). Pretreatment with synbiotics resulted in significantly less activation of SMAD3/4 compared to the cells treated with *L. acidophilus* alone, suggesting that the combination of inulin and *L. acidophilus* has additional effects on TGF-β/SMAD signaling. In addition, the cells pretreated with TGF-β antibody modestly diminished the effects of *L. acidophilus* on SMAD3/4 and NF-κB transcriptional activity (Fig. 5), supporting the hypothesis that *L. acidophilus* activates TGF-β signaling to suppress inflammation caused by *S. typhimurium* in intestinal cells.

## Discussion

Human immunity plays an important role in the development of more serious clinical diseases after *S. typhimurium* infection because of increased pro-inflammatory cytokine expression in the intestine [25]. *S. typhimurium* infection activates NF-κB in intestinal epithelium cells and subsequently up-regulates gene transcription of pro-inflammatory cytokines, such as IL-8 [26] and TNF-α [22]. The present study demonstrates that *S. typhimurium* infection induces TNF-α and IL-8 pro-inflammatory cytokine expression in human intestinal Caco-2 cells. The previous study demonstrated that *L. acidophilus* pretreatment decreases *S. typhimurium* induced inflammation [7]. The finding is consistent with the results showing that anti-inflammatory effects are achieved by probiotics in clinical setting [27]. In normal intestinal mucosal cells, the TGF-β signal may negatively regulate NF-κB activation by stimulating the negative regulator, IκBα [16]. Silencing SMAD7 can restore TGFβ/SMAD3 signaling and result in the suppression of inflammatory cytokine production in patients with inflammatory bowel diseases [28]. Our results reveal that *L. acidophilus* induces TGF-β/MIR21 expression and down-regulates SMAD7 expression, which may decrease NF-κB-activated inflammation caused by *S. typhimurium* in human intestinal Caco-2 cells.

A prebiotic was first defined as a non-digestible food ingredient that beneficially affects the host by selectively stimulating the growth and/or activity of one or a limited number of bacteria in the colon and improving host health [29]. Preliminary research on prebiotics demonstrated potential effects on calcium and other mineral absorption [30], immune system effectiveness [31], bowel pH, reduction of colorectal cancer risk [32], and inflammatory bowel disorders, such as Crohn's disease and ulcerative colitis [33]. Additionally, Roberfroid stated that only two particular prebiotics then fully meet this definition, including trans-galactooligosaccharide and inulin [29]. Inulin, a long-chain prebiotic, stimulates the growth of probiotics [29]. Our results showed that combination of probiotic and inulin as synbiotic had a suppressive effect on the *S. typhimurium*-induced inflammatory cytokine production in human intestinal Caco-2 cells, including IL-8 and TNF-α. However, synbiotics showed a smaller effect on TGF-β and MIR21 expression, and SMAD3/4 transcriptional activity compared to the cells treated with *L. acidophilus* alone during infection with *S. typhimurium.* Previous report indicates that prebiotic oligosaccharides reduce proinflammatory cytokines in intestinal Caco-2 cells via activation of PPARγ and peptidoglycan recognition protein 3 [34]. These results suggest that inulin may trigger additional signaling events to prevent *S. typhimurium*-induced inflammation in a TGF-β independent manner.

*S. typhimurium* is an intracellular bacterium that enters cells after 2 h of infection. We infected Caco-2 cells with *S. typhimurium* for only 1 h and recovered the cells with media containing gentamicin. Therefore, the effects on the NF-κB activation and inflammatory cytokines induction might be resulted from lipopolysaccharides of *S. typhimurium.* On the other hand, our current study showed that *L. acidophilus* significantly induced TGF-β/SMAD activity in intestinal cells. In addition to TGF-β/MIR21 regulation on SMAD7 expression, *L. acidophilus* inactivates Jak1/Stat1 signaling to decrease SMAD7 expression, suggesting that Jak1/Stat1 may be involved in the regulation of *L. acidophilus* on *S. typhimurium*-induced SMAD7 expression [9]. It is necessary to further elucidate the detailed mechanisms of TGF-β/SMAD regulation in human intestinal cells treated with *L. acidophilus*.

Microbes play a pivotal role in intestinal homeostasis, such as the uptake of nutrients and the generation of essential elements. However, pathogen infection may disrupt the homeostasis of intestinal microbes and cause acute gastroenteritis or chronic inflammatory diseases. Numerous clinical reports show that probiotics play beneficial roles on reducing incidence of gastrointestinal infectious diseases, including acute diarrhea, necrotizing enterocolitis and death [35–37]. Our study showed that TGF-β1/MIR21 signaling pathway may be involved in the suppressive effects of *L. acidophilus* on inflammation in intestinal cells during *S. typhimurium* infection. Previous reports also indicate that prebiotics activate immune system in animals during enteric pathogen infection [31]. However, our current study show that prebiotic (inulin) has no effect on diminishing inflammation in intestinal cells during *S. typhimurium* infection, suggesting inulin may preferentially modulate immune cells to reduce inflammation *in vivo*. Although the cell culture model may not be able to precisely reflect actual infection due to complicated nature of microbiota and host defense system *in vivo*, our study provide a potential mechanism of *L. acidophilus* on inflammation suppression in intestinal cells during *S. typhimurium* infection.

## Conclusion

Our current study indicates that *L. acidophilus* induces TGF-β/MIR21 expression to reduce NF-κB-activated inflammation caused by *S. typhimurium* in human intestinal Caco-2 cells. The TGF-β/MIR21 expression might be used as a marker to evaluate anti-inflammatory effects of other strains of *Lactobacillus*, which requires further study to determine the potential application.

## Additional files

Additional file 1: Figure S1. Time required for *S. typhimurium* to enter host cells. Human intestinal Caco-2 cells were incubated with *S. typhimurium* (sal, 1×10^7^ CFU per well) for 1, 2, or 3 h in a six-well plate. The cells were subsequently washed with PBS, and the medium was replaced with medium containing with antibiotics for 6 h. The cells were then lysed with 1 % Triton X-100 (in PBS) to obtain the intracellular *S. typhimurium*. Then, the lysates were incubated overnight on LB agar to determine the time required for *S. typhimurium* to enter cells. (TIFF 234 kb) Additional file 2: Figure S2. Effect of *L. acidophilus* on SMAD3/4 transcriptional activity with active or inactive *S. typhimurium*. Human intestinal Caco-2 cells were transfected with luciferase reporter plasmid containing the promoter of the SMAD-binding site or CMV promoter overnight. The cells were then pretreated with *L. acidophilus* (MOI: 20) 1 h prior to infection with *S. typhimurium* (MOI: 10) or UV-inactivated *S. typhimurium* (MOI: 10) in antibiotic-free DMEM for 1 h at 37 °C. Then, the cells were washed twice with PBS and added to medium containing D-luciferin to monitor SMAD activity. The cells were washed twice with PBS, added to DMEM medium containing D-luciferin and antibiotics for signal measurement with a Luminometer. Data were analyzed with Prism 5, and the results are shown as the means ± SEM from three independent experiments. (TIFF 100 kb)

## Abbreviations

*S. typhimurium*: *Salmonella typhimurium*
*L. acidophilus*: *Lactobacillus acidophilus*
TGF-β: Transforming growth factor beta
NF-κB: Nuclear Factor-kappa B
CFU: Colony-forming unit
IL-8: Chemokine (C-X-C motif) ligand 8
TNF-α: Tumor necrosis factor alpha
SMAD7: SMAD family member 7
MIR21: MicroRNA 21
