# Supplementary figures and images for: Lactobacillus acidophilus attenuates Salmonella-induced intestinal inflammation via TGF-β signaling
Source: BMC Microbiol. 2015 Oct 7;15:203. doi: 10.1186/s12866-015-0546-x (PMC4596496; doi:10.1186/s12866-015-0546-x)

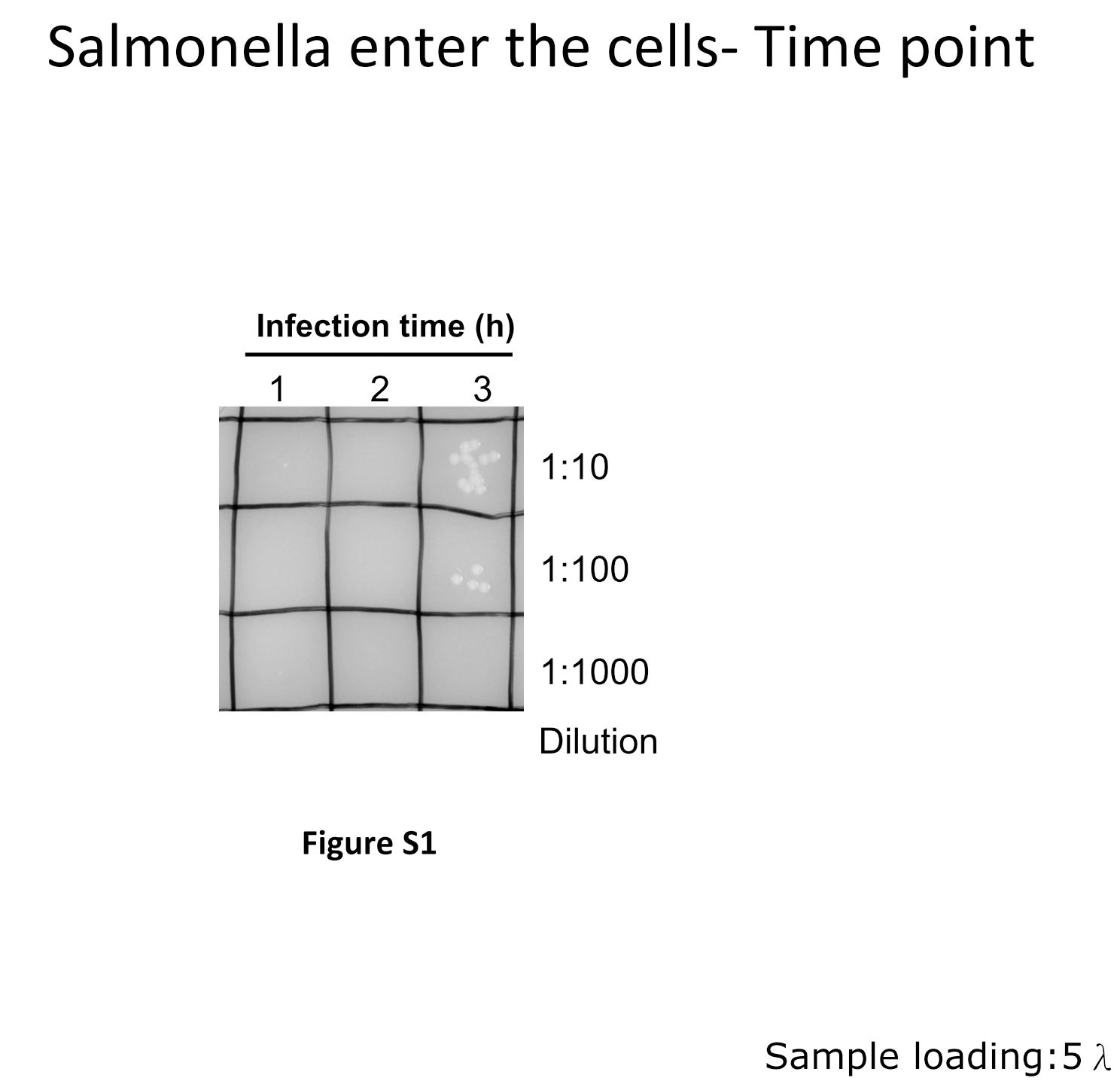

Supplement: Additional file 1: Figure S1. — Time required for S. typhimurium to enter host cells. Human intestinal Caco-2 cells were incubated with S. typhimurium (sal, 1×10^7^ CFU per well) for 1, 2, or 3 h in a six-well plate. The cells were subsequently washed with PBS, and the medium was replaced with medium containing with antibiotics for 6 h. The cells were then lysed with 1 % Triton X-100 (in PBS) to obtain the intracellular S. typhimurium. Then, the lysates were incubated overnight on LB agar to determine the time required for S. typhimurium to enter cells. (TIFF 234 kb) [file 12866_2015_546_MOESM1_ESM.tif]

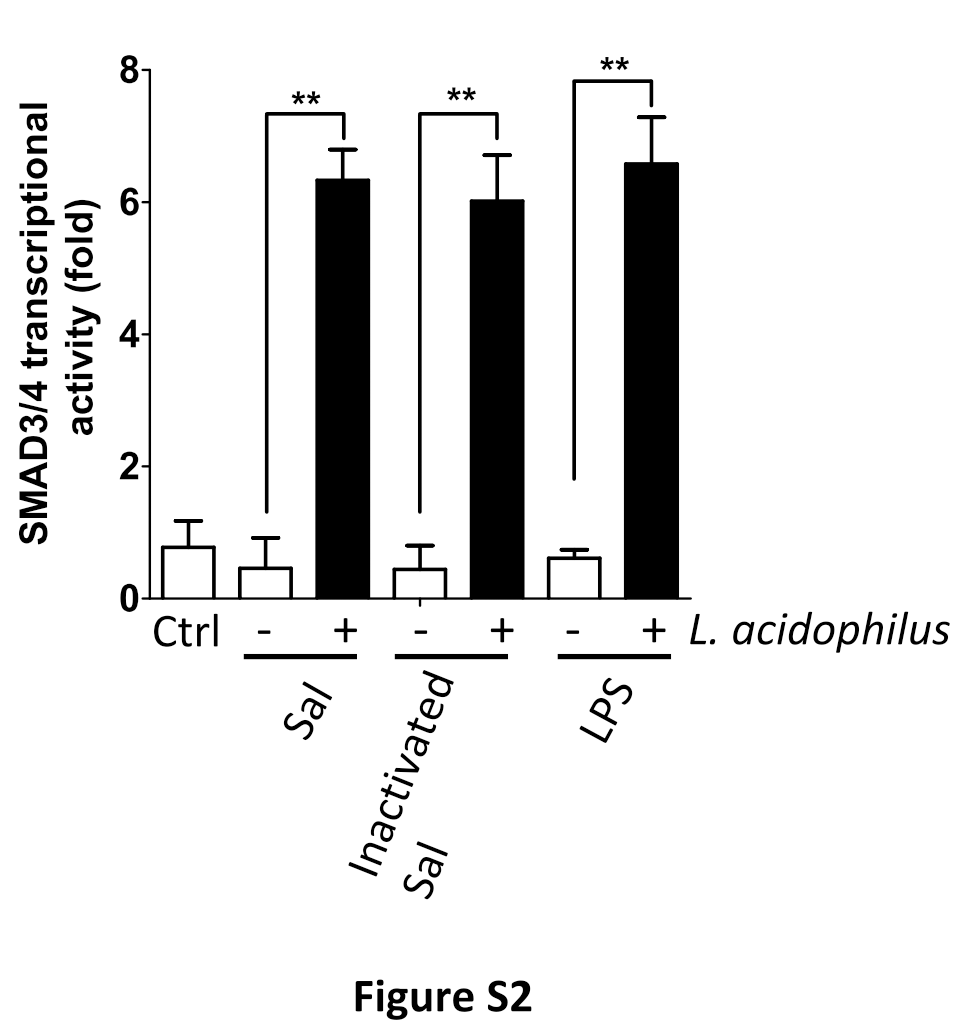

Supplement: Additional file 2: Figure S2. — Effect of L. acidophilus on SMAD3/4 transcriptional activity with active or inactive S. typhimurium. Human intestinal Caco-2 cells were transfected with luciferase reporter plasmid containing the promoter of the SMAD-binding site or CMV promoter overnight. The cells were then pretreated with L. acidophilus (MOI: 20) 1 h prior to infection with S. typhimurium (MOI: 10) or UV-inactivated S. typhimurium (MOI: 10) in antibiotic-free DMEM for 1 h at 37 °C. Then, the cells were washed twice with PBS and added to medium containing D-luciferin to monitor SMAD activity. The cells were washed twice with PBS, added to DMEM medium containing D-luciferin and antibiotics for signal measurement with a Luminometer. Data were analyzed with Prism 5, and the results are shown as the means ± SEM from three independent experiments. (TIFF 100 kb) [file 12866_2015_546_MOESM2_ESM.tif]
